# The Association of a Mindfulness-Based Stress Reduction Nursing Model With Quality of Life in Patients With Chronic Tonsillitis Complicated by Anxiety Disorder: A Retrospective Study

**DOI:** 10.62641/aep.2272

**Published:** 2026-10-15

**Authors:** Xiaodan Zhang, Miao Wang, Qin Fu

**Affiliations:** ^1^Department of Otorhinolaryngology-Head and Neck Surgery, Yingshang County People's Hospital, 236200 Fuyang, Anhui, China

**Keywords:** mindfulness, tonsillitis, anxiety disorders, quality of life, sleep quality

## Abstract

**Background:**

To examine the association between a mindfulness-based stress reduction (MBSR) nursing model and changes in quality of life in patients with chronic tonsillitis complicated by anxiety disorder.

**Methods:**

This retrospective study analyzed propensity score-matched data from 110 patients (55 per group) with chronic tonsillitis and comorbid anxiety disorder treated at Yingshang County People’s Hospital from February 2023 to October 2025. Based on nursing records, patients received either routine nursing or the MBSR nursing model. Pharyngeal symptom scores, Generalized Anxiety Disorder-7 (GAD-7) scores, Mindful Attention Awareness Scale (MAAS) scores, Hamilton Anxiety (HAMA) Scale scores, Pittsburgh Sleep Quality Index (PSQI) scores, and World Health Organization Quality of Life Brief Version (WHOQOL-BREF) scores were extracted and compared. Polysomnography parameters were also analyzed. Exploratory encephalofluctuogram (EFG) data are presented in the Supplementary Material.

**Results:**

Post-nursing, both groups showed significantly lower scores for all eight pharyngeal symptoms and lower GAD-7, HAMA, and PSQI scores and higher MAAS scores compared to pre-nursing values (*p* < 0.05). The MBSR group demonstrated significantly lower pharyngeal symptom scores, lower GAD-7, HAMA, and PSQI scores, and higher MAAS scores than the routine nursing group (*p* < 0.05), along with significantly higher scores across all WHOQOL-BREF domains (*p* < 0.05). Polysomnography revealed significantly shorter sleep latency, fewer micro-arousals, and a higher proportion of rapid eye movement sleep in the MBSR group (*p* < 0.05). Exploratory EFG observations are presented in the Supplementary Material.

**Conclusions:**

In this retrospective analysis, the MBSR nursing model was associated with lower anxiety levels, higher mindful attention awareness, better sleep quality, and better overall quality of life in patients with chronic tonsillitis complicated by anxiety disorder. Exploratory EFG observations are hypothesis-generating only and require validation with established methods. These findings warrant further investigation in prospective studies.

## Introduction

Chronic tonsillitis is primarily characterized by persistent and recurrent pharyngeal discomfort, with symptoms such as sore throat, foreign body sensation, halitosis, and dysphagia often fluctuating in intensity [1]. These symptoms carry a strong somatic focus, easily triggering threatening interpretations and hypervigilance, thereby promoting worry, rumination, sleep impairment, and functional limitations. This creates a cycle where physical symptoms and anxiety disorder mutually reinforce each other [2]. Within standard nursing pathways, while basic support is provided through health education, medication guidance, and symptom monitoring, there is often insufficient attention to core psychological processes such as attentional bias, decentering ability, and emotional non-reactivity [3]. The mindfulness-based stress reduction (MBSR) nursing model, centered on present-moment awareness, non-judgmental acceptance, and attentional regulation training, has been proposed to alleviate symptom-related catastrophic interpretations and avoidance behaviors by enhancing bodily awareness and cognitive defusion capabilities, potentially improving sleep and quality of life [4]. Existing mindfulness-based approaches predominantly target general populations or chronic conditions like cancer, with relatively limited development of specialized nursing pathways and combined objective outcome evaluations specifically for chronic tonsillitis with comorbid anxiety [5, 6]. Therefore, this study utilized a retrospective design to extract data on patients with chronic tonsillitis and anxiety disorder, employed propensity score matching (PSM) to ensure intergroup balance, and compared documented outcomes between MBSR nursing and routine nursing. As an exploratory component, encephalofluctuogram (EFG) data were also extracted and are presented in the Supplementary Material. EFG is not a validated clinical tool for measuring neurotransmitter levels or brain activity; these data are purely hypothesis-generating and should not be considered a main outcome.

## Materials and Methods

### General Information

This study employed a retrospective design. The sample size was estimated based on the expected between-group difference in the Generalized Anxiety Disorder-7 (GAD-7) score. Based on a systematic review and meta-analysis of mindfulness-based treatments for anxiety disorders, which reported effect sizes generally in the moderate range, a standardized mean difference (Cohen’s d) in GAD-7 scores between the MBSR nursing group and the routine nursing group was estimated to be approximately 0.60 [7]. With a two-sided significance level of α = 0.05 and a statistical power of 1-β = 0.80, the sample size calculation for comparing two independent means indicated that 45 patients were required per group.

A review of the hospital electronic medical record system identified a total of 237 patients with a recorded diagnosis of chronic tonsillitis complicated by anxiety disorder between February 2023 and October 2025. Following a case-by-case review based on predefined inclusion and exclusion criteria, patients were excluded for the following documented reasons: severe comorbid mental illness (n = 8), significant cognitive impairment (n = 8), severe comorbid somatic disease affecting quality of life (n = 10), pregnancy or lactation (n = 5), major life stress event within the past 3 months (n = 3), and other comorbid pharyngeal diseases (n = 4). Ultimately, data from 199 eligible patients were included in the analysis. Based on the nursing pathway, nursing records, and related implementation documentation extracted from the electronic medical record system, patients were categorized into two groups: 105 in the routine nursing group and 94 in the MBSR nursing group. PSM was performed to balance baseline characteristics between the two groups. The propensity score was calculated using a logistic regression model that included age, body mass index (BMI), disease duration, tonsil grade, smoking status (yes/no), alcohol consumption (yes/no), and education level as covariates. Nearest-neighbor matching without replacement was performed using a caliper width set to 0.2 of the standard deviation of the logit of the propensity score in a 1:1 ratio. After matching, the standardized mean differences (SMD) for all covariates were <0.1, indicating adequate balance between the groups. Following matching, 55 patients were included in each group. According to the extracted records, the routine nursing group received the standard nursing model, and the MBSR nursing group received the MBSR nursing model. This study was approved by the Ethics Committee of Yingshang County People’s Hospital (Approval No.: YS20260202603), and all study procedures were conducted in accordance with the Declaration of Helsinki, with documentation of patients’ informed consent and signed consent forms in the medical records.

### Inclusion and Exclusion Criteria

Inclusion criteria: (1) Documented diagnosis meeting criteria for grade II or III chronic tonsillitis according to Otorhinolaryngology Head and Neck Surgery [8], with a recorded disease duration ≥1 year and acute exacerbations ≥5 times per year; (2) Documented diagnosis meeting criteria for Generalized Anxiety Disorder according to the Diagnostic and Statistical Manual of Mental Disorders (DSM-5) [9], with a recorded disease duration ≥6 months and a GAD-7 score ≥10 in the medical record; the cutoff score of ≥10 was used to indicate moderate or more severe anxiety, as supported by the original validation study [10]; (3) Recorded age 18 to 65 years, regardless of gender; (4) Medical record documentation indicating that the patient received a complete course of either the “MBSR nursing model” or the “routine nursing model”; (5) Availability of complete core evaluation data in the medical records. Exclusion criteria: (1) Documented comorbid severe mental illnesses such as schizophrenia, bipolar disorder, major depressive disorder, substance abuse disorder, severe personality disorders; (2) Documented significant cognitive impairment; (3) Documented comorbid other somatic diseases severely affecting quality of life, such as malignant tumors, severe heart failure, gastrointestinal diseases, chronic obstructive pulmonary disease, liver or kidney insufficiency, rheumatoid arthritis; (4) Documented pregnancy or breastfeeding women; (5) Documented major life stress events within the past 3 months; (6) Documented comorbid other pharyngeal diseases, such as chronic pharyngitis, diphtheria, pharyngeal tumors.

### Nursing Models

Based on the extracted records, the nursing period for both groups was 4 weeks. All nursing measures were documented as being implemented according to the established nursing pathways of the ward. For the MBSR nursing group, the detailed intervention records (e.g., daily practices, online sessions, specific techniques) were documented using program-specific structured templates—such as teaching checklists, sign-in forms, WeChat mini-program check-in records, and weekly interview forms—which were stored together with the medical records. This allowed retrospective extraction of granular intervention data beyond what is typically found in routine clinical notes. Documentation of the nursing process included nursing record sheets, specialized training sign-in forms, WeChat mini-program check-in records, online group meeting records, and weekly interview records. All records indicated that enrolled participants completed the corresponding nursing period, and the completion rate of core training modules was ≥80%.

Routine Nursing Group: Extracted records indicated use of a standardized nursing pathway for chronic tonsillitis [11, 12, 13]. (1) Upon admission, records showed assessment of disease understanding and health education were conducted, covering disease mechanisms, medication plans, and surgical indications. (2) During hospitalization, records showed that vital signs and changes in pharyngeal symptoms were monitored. Symptomatic treatments such as anti-infective and analgesic therapies were administered per physician’s orders. Guidance on a light diet and oral hygiene maintenance was provided. (3) Upon discharge, records indicated medication instructions, reminders for follow-up appointments, and routine psychological reassurance were given.

MBSR Nursing Group: Extracted records indicated that the MBSR nursing model was implemented. The following description of the nursing content is based on the standardized pathway documented in the medical and nursing implementation records. (1) Phase 1: Explanation of Core Mechanisms and Attention Anchoring Training (Early Hospitalization) [4, 14, 15]. (a) Mindfulness Cognitive Introduction: Records indicated structured mindfulness education was delivered using multimedia presentations combined with experiential teaching to explain the core mechanisms of mindfulness (attentional regulation, body awareness, decentering). Emphasis was placed on explaining the neurobiological mechanisms of the anxiety-inflammation interaction, guiding patients to establish awareness of the symptom-emotion-cognition triangle. Distribution of a Mindfulness-Based Stress Reduction Self-Practice Manual and establishment of a digital mindfulness check-in platform based on a WeChat mini-program were documented. (b) Breath Anchoring and Throat-Specific Body Scan: Records indicated guided mindfulness training was conducted twice daily, 20 minutes per session. A modified self-developed body scan was performed in the morning, adding a refined throat area awareness segment (5 minutes) to the classic head-to-toe awareness process. Patients were guided to observe the physical properties (temperature, texture, intensity changes) of pharyngeal discomfort with a non-judgmental attitude, establishing a response pattern of awareness, labeling, and acceptance to replace avoidance or catastrophic cognition. Breath anchoring training was conducted in the evening using a 4-7-8 breathing rhythm (inhale for 4 seconds, hold breath for 7 seconds, exhale for 8 seconds). (c) Mindfulness Skills Training for Symptom Response: For acute symptoms like sore throat and foreign body sensation, records indicated implementation of the S.T.O.P. (Stop–Take a breath–Observe–Proceed) short practice derived from mindfulness training was implemented. When symptoms occurred, patients were instructed to pause their current activity, take three deep breaths, then observe symptom-related thoughts, emotions, and physical sensations from an observer’s perspective, labeling them as “this is a thought about pain” rather than fact, thereby reducing symptom catastrophizing. Concurrently, a mindfulness cold compress technique was documented as being introduced. While applying a cold compress to the tonsil area, patients were guided to focus attention on the physical attributes of the cold sensation (coolness, pressure, diffusion range) to reduce sustained focus on the pain experience and decrease pain sensitivity.

(2) Phase 2: Integration of Mind-Body Awareness and Strengthened Contextual Symptom Response (Mid-to-Late Hospitalization/Pre-Discharge) [16, 17, 18]: (a) Digital Mindfulness Group: Records indicated a 90-minute online group mindfulness session was held weekly (via Zoom/Tencent Meeting platform) using a co-facilitator model (mindfulness instructor + otorhinolaryngology (ENT) specialist nurse). The session structure included: 30 minutes of thematic mindfulness practice (mindful yoga, loving-kindness meditation, sound awareness meditation), 40 minutes of experience sharing and symptom discussion, and 20 minutes of Q&A guidance. A mindfulness partner pairing mechanism was documented, where patients paired up to send each other daily voice messages with mindful greetings and share their daily awareness experiences, strengthening social support networks. (b) Mindful Eating and Swallowing Training: Aimed at swallowing discomfort and halitosis, mindful eating training was documented as being implemented. Patients were asked to designate one meal per day as a “mindful meal”, following a five-step method: observe–smell–take a small bite/taste–chew with awareness–sense swallowing, extending the total meal duration to 20–30 minutes. Special attention was given to sensing the tension in the throat muscles during swallowing, guiding patients to consciously relax the pharyngeal muscles to establish a new swallowing pattern. Simultaneously, mindful gargling with warm saline was practiced, during which patients were guided to be aware of the liquid’s temperature, taste layers, and tactile sensation on the pharyngeal mucosa, extending gargling time to 3 minutes to replace mechanical mouth rinsing. (c) Pre-Sleep Cognitive Defusion Techniques: Records indicated patients were guided using the “thought train” metaphor (viewing anxious thoughts as trains passing through a station, observing them without boarding), accompanied by a body scan audio recording (specially recorded with a slowed pace of 90 words per minute). A “digital sunset” rule was documented, requiring electronic devices to be turned off one hour before sleep, and mindful sitting by candlelight was practiced to promote sleep onset through visual focusing training.

(3) Phase 3: Consolidation of Routine Mindfulness Practice and Establishment of a Relapse Prevention Plan (Completed Pre-Discharge) [14, 17, 19, 20]. (a) Consolidation of Self-Practice Ability and Establishment of a Post-Discharge Routine Implementation Checklist: Records indicated dependence on guided audio was gradually reduced, and unguided mindful walking and mindful daily activities (e.g., mindful brushing, mindful showering) were encouraged. A mindfulness emergency card was documented as being created, listing core skills like the STOP technique and the 3-minute breathing space on a portable card for immediate reference during acute symptom flare-ups or escalating anxiety. (b) Relapse Prevention and Self-Efficacy Enhancement: Records indicated that integrated mindfulness-based cognitive therapy techniques were used to identify individual early warning signs of anxiety recurrence and develop personalized coping scripts. Weekly interviews were conducted to inquire about symptom changes, check practice adherence, and reinforce practice points. A patient-led mindfulness experience-sharing community was documented as being established to encourage continued mutual support after the nursing period concluded.

(4) Quality Control and Assurance: According to records, all mindfulness-based nursing was delivered jointly by psychiatric specialist nurses with MBSR teacher certification and ENT head nurses. All nursing staff involved had documentation of receiving standardized training prior to the nursing period. A standardized mindfulness teaching checklist and nursing implementation form were documented as being used for each session to structurally record training content, duration, patient attendance, home practice completion, and weekly interview completion, ensuring consistency of delivery. During retrospective data extraction, two researchers reviewed the aforementioned original records to verify nursing fidelity, adherence, and exposure duration; patients with incomplete records or those whose completion status could not be confirmed were not classified as having received the MBSR nursing exposure.

### Outcome Measures and Assessment Methods

#### Baseline Data

Baseline data were extracted from inpatient medical records and admission assessment forms. Extracted variables included gender, age, body mass index (BMI), education level (junior high school or below, high school/vocational school, bachelor’s degree or above), smoking history (yes/no), and alcohol consumption history (yes/no). Disease-related data, including chronic tonsillitis duration (years) and tonsil grading (grade II/III), were also extracted.

#### Scoring Criteria

All scale scores were extracted from the medical records; the scales had been administered as part of routine care by uniformly trained otorhinolaryngology nurses. The following describes the standardized, validated instruments used for assessment. (1) Pharyngeal Symptom Score: This scale was developed by our research team, referencing the symptom assessment framework and item pool of the adult chronic pharyngitis symptom scale developed by Müller *et al*. [21], to assess common pharyngeal symptoms of chronic tonsillitis, including dry throat, itchy throat, pharyngeal foreign body sensation, pharyngeal burning sensation, halitosis, sore throat, expectoration, and expectoration of tonsillar debris. Each item was scored from 0 to 6, with higher scores indicating more severe symptoms. In the present sample, the scale demonstrated good internal consistency (Cronbach’s α = 0.86) . (2) GAD-7 Score [22]: Consists of 7 items, each scored from 0 to 3, with a total score ranging from 0 to 21. The cut-off value was 10, with higher scores indicating greater anxiety severity. (3) Hamilton Anxiety Scale (HAMA) [23]: Consists of 14 items, with a total possible score of 56. Scores >7 suggest possible anxiety, >14 indicates definite anxiety, >21 indicates significant anxiety, and >29 indicates severe anxiety [24]. (4) Pittsburgh Sleep Quality Index (PSQI) [25]: Comprises 7 components, each scored from 0 to 3, with a total score ranging from 0 to 21. Higher scores indicate poorer sleep quality. (5) Mindful Attention Awareness Scale (MAAS) [26]: Consists of 15 items, each scored from 1 to 6, with a total score ranging from 15 to 90. Higher scores indicate higher levels of mindful attention awareness. (6) World Health Organization Quality of Life Brief Version (WHOQOL-BREF) [27]: Consists of 26 items; items 1 and 2 are global items assessing overall quality of life and general health and are not included in domain calculations. The scale comprises the physical (score range: 7–35), psychological (score range: 6–30), social relationship (score range: 3–15), and environmental (score range: 8–40) domains. Items are scored from 1 to 5 using raw scores, with higher scores indicating better quality of life.

#### Encephalofluctuogram (EFG) Ultra-Slow Wave Relative Power Values

Data on EFG ultra-slow wave relative power values [28] were extracted from prior clinical assessments documented in the medical records. It is important to note that EFG is not a validated clinical tool for measuring neurotransmitter levels or brain activity. The analysis was based on EEG ultra-slow fluctuation theory, and the corresponding frequency bands are named according to conventions in this exploratory field; these results should be interpreted purely as exploratory electrophysiological phenomena and do not represent direct measurements of neurotransmitter concentrations. The extracted data were derived from recordings made with sixteen-lead electrodes placed according to the international 10/20 system. Patients were documented as being awake, with eyes closed, in a quiet state, while EEG signals were continuously recorded for 10 minutes. During acquisition, the environment was kept quiet and with appropriate lighting, and potential interfering factors such as body movements and eye blinks were recorded. After acquisition, raw signals underwent artifact rejection and quality control (removing segments with significant electromyography, electrooculography, and motion artifacts). Power spectrum analysis of ultra-slow waves was performed on the validated signals to calculate the relative power values for each target frequency band. This study examined extracted relative power values for frequency bands designated in the relevant literature as 1 mHz, 2 mHz, 4 mHz, 5 mHz, 7 mHz, and 11 mHz. These designations are conventional labels used in ultra-slow wave research and do not imply direct correspondence to specific neurotransmitter systems. Given the exploratory nature of EFG and the lack of validation as a clinical measurement tool, the EFG results are presented only in the **Supplementary Table 1** and are not discussed as primary findings.

#### Polysomnography Results

Data on sleep latency, percentage of rapid eye movement (REM) sleep, and number of micro-arousals were extracted from polysomnography reports performed before and after the nursing period using an SF-A9 polysomnography system (Hunan Viamax Medical Technology Co., Ltd., Xiangxiezhun 20192070393). Micro-arousals were defined as the number of micro-arousals per hour of sleep, according to the American Academy of Sleep Medicine (AASM) scoring criteria [29].

### Statistical Methods

SPSS 25.0 (IBM Corp., Armonk, NY, USA) was used to analyze data organized in Excel. The normality of continuous data distribution was assessed using the Shapiro-Wilk test. Measurement data conforming to normal distribution were expressed as mean ± standard deviation ($\bar{x}$ ± s). Paired t-tests were used for both within-group comparisons (pre- vs. post-nursing) and between-group comparisons (matched pairs after PSM). Non-normally distributed quantitative data were described using median (interquartile range) [M (P25-P75)], and between-group comparisons were performed using the paired-sample Wilcoxon signed-rank test. Count data are expressed as n (%), and the McNemar test was used for comparison between matched groups. All tests were two-sided, and a *p*-value < 0.05 was considered statistically significant. Scale data entry was performed independently by two individuals with cross-checking to ensure data accuracy.

## Results

### Comparison of Baseline Data Between the Two Groups

No statistically significant differences were found in the extracted baseline characteristics such as gender, age, smoking status, or alcohol consumption status between the two groups after matching (*p* > 0.05). See Tables 1 and 2.

**Table 1.** **Comparison of baseline data before matching between the two groups**.

Baseline data		Routine nursing group (n = 105)	MBSR nursing group (n = 94)	χ^2^/t value	*p* value
Gender [n(%)]				0.009	0.926
	Male	61 (58.10)	54 (57.45)		
	Female	44 (41.90)	40 (42.55)		
Age (years)		45.20 ± 6.03	43.89 ± 8.51	1.263	0.208
BMI(kg/m^2^)		23.56 ± 2.33	23.41 ± 2.61	0.428	0.669
Disease duration (years)		1.80 ± 0.41	1.83 ± 0.48	–0.475	0.635
Smoking [n (%)]				4.431	0.035
	Yes	37 (35.24)	47 (50.00)		
	No	68 (64.76)	47 (50.00)		
Alcohol consumption [n (%)]				4.237	0.040
	Yes	26 (24.76)	36 (38.30)		
	No	79 (75.24)	58 (61.70)		
Tonsil grading [n (%)]				0.303	0.582
	Grade II	72 (68.57)	61 (64.89)		
	Grade III	33 (31.43)	33 (35.11)		
Education level [n (%)]				0.641	0.726
	Junior high school or below	18 (17.14)	18 (19.15)		
	High school/Vocational school	54 (51.43)	43 (45.74)		
	Bachelor’s degree or above	33 (31.43)	33 (35.11)		

Note: BMI, body mass index.

**Table 2.** **Comparison of baseline data after matching between the two groups**.

Baseline characteristic		Routine nursing group (n = 55)	MBSR nursing group (n = 55)	Statistic	*p* value
Gender [n (%)]				McNemar exact *p*	0.727
	Male	28 (50.91)	30 (54.55)		
	Female	27 (49.09)	25 (45.45)		
Age (years)		42.12 ± 5.23	41.87 ± 5.39	0.309	0.758
BMI (kg/m^2^)		23.24 ± 2.06	23.30 ± 2.14	0.178	0.859
Disease duration (years)		1.85 ± 0.36	1.81 ± 0.42	0.597	0.553
Smoking [n (%)]				McNemar exact *p*	0.388
	Yes	18 (32.73)	22 (40.00)		
	No	37 (67.27)	33 (60.00)		
Alcohol consumption [n (%)]				McNemar exact *p*	0.508
	Yes	14 (25.45)	11 (20.00)		
	No	41 (74.55)	44 (80.00)		
Tonsil grading [n (%)]				McNemar exact *p*	0.727
	Grade II	38 (69.09)	34 (61.82)		
	Grade III	17 (30.91)	21 (38.18)		
Education level [n (%)]				McNemar exact *p*	1.000
	Junior high school or below	9 (16.36)	10 (18.18)		
	High school/Vocational school	27 (49.09)	25 (45.45)		
	Bachelor’s degree or above	19 (34.55)	20 (36.36)		

Note: BMI, body mass index. For categorical variables, the exact *p* value from McNemar’s test is provided.

### Comparison of Pharyngeal Symptom Scores Between the Two Groups

Analysis of extracted data showed no significant differences were observed in pharyngeal symptom scores between the two groups before nursing (*p* > 0.05). Following the nursing period, extracted data indicated that scores for dry throat, itchy throat, pharyngeal foreign body sensation, pharyngeal burning sensation, halitosis, sore throat, expectoration, and expectoration of tonsillar debris were significantly lower in both groups compared to their respective pre-nursing values (*p* < 0.05), and the MBSR nursing group had significantly lower scores for these eight pharyngeal symptoms than the routine nursing group (*p* < 0.05). See Table 3.

**Table 3.** **Comparison of pharyngeal symptom scores between the two groups ($\bar{x}$ ± s) point**.

Symptom	Time	Routine nursing group (n = 55)	MBSR nursing group (n = 55)	T value	*p* value
Dry throat	Before nursing	4.25 ± 0.54	4.19 ± 0.63	0.536	0.593
	After nursing	2.31 ± 0.50*	1.73 ± 0.45*	6.394	<0.001
Itchy throat	Before nursing	4.11 ± 0.48	4.20 ± 0.46	–1.004	0.318
	After nursing	1.96 ± 0.23*	1.55 ± 0.50*	5.525	<0.001
Pharyngeal foreign body sensation	Before nursing	4.30 ± 0.52	4.25 ± 0.56	0.485	0.628
	After nursing	2.07 ± 0.33*	1.62 ± 0.49*	5.649	<0.001
Pharyngeal burning sensation	Before nursing	4.15 ± 0.36	4.20 ± 0.44	–0.652	0.516
	After nursing	1.96 ± 0.28*	1.54 ± 0.50*	5.436	<0.001
Halitosis	Before nursing	4.26 ± 0.44	4.32 ± 0.47	–0.691	0.491
	After nursing	2.11 ± 0.31*	1.61 ± 0.52*	6.125	<0.001
Sore throat	Before nursing	3.96 ± 0.52	4.05 ± 0.45	–0.971	0.334
	After nursing	1.87 ± 0.30*	1.65 ± 0.48*	2.882	0.005
Expectoration	Before nursing	4.56 ± 0.51	4.51 ± 0.58	0.480	0.632
	After nursing	2.23 ± 0.45*	1.61 ± 0.49*	6.911	<0.001
Expectoration of tonsillar debris	Before nursing	3.47 ± 0.50	3.51 ± 0.50	–0.420	0.676
	After nursing	1.84 ± 0.50*	1.57 ± 0.51*	2.804	0.006

Note: Compared with pre-nursing status, *p < 0.05.

### Comparison of GAD-7, HAMA, and PSQI Scores Between the Two Groups

Analysis of extracted data showed no significant differences were found in GAD-7, HAMA, and PSQI scores between the two groups before nursing (*p* > 0.05). Following the nursing period, extracted data indicated that GAD-7, HAMA, and PSQI scores were significantly lower in both groups compared to before nursing (*p* < 0.05), and the MBSR nursing group had significantly lower scores on all three scales compared to the routine nursing group (*p* < 0.05). See Table 4.

**Table 4.** **Comparison of GAD-7 scores, HAMA scores, and PSQI scores between the two groups ($\bar{x}$ ± s) point**.

Group	n	GAD-7 score		HAMA score		PSQI score	
		Before nursing	After nursing	Before nursing	After nursing	Before nursing	After nursing
Routine nursing group	55	14.58 ± 2.17	11.02 ± 2.08*	21.41 ± 3.96	14.26 ± 2.85*	12.20 ± 2.86	9.64 ± 1.87*
MBSR nursing group	55	14.63 ± 2.53	8.73 ± 1.47*	21.56 ± 4.01	10.04 ± 2.51*	12.11 ± 2.93	7.84 ± 1.51*
t value		–0.111	6.668	–0.197	8.241	0.163	5.554
*p* value		0.912	<0.001	0.844	<0.001	0.871	<0.001

Note: Compared with pre-nursing status, *p < 0.05; GAD-7, Generalized Anxiety Disorder-7; HAMA, Hamilton Anxiety Scale; PSQI, Pittsburgh Sleep Quality Index.

### Comparison of MAAS Scores Between the Two Groups

Analysis of extracted data showed no significant difference was found in MAAS scores between the two groups before nursing (*p* > 0.05). Following the nursing period, extracted data indicated that MAAS scores increased significantly in both groups compared to before nursing (*p* < 0.05), and the MBSR nursing group had a significantly higher MAAS score than the routine nursing group (*p* < 0.05). See Table 5.

**Table 5.** **Comparison of MAAS scores between the two groups ($\bar{x}$ ± s) point**.

Group	n	MAAS score	
		Before nursing	After nursing
Routine nursing group	55	47.74 ± 4.23	54.72 ± 4.93*
MBSR nursing group	55	48.04 ± 5.12	59.84 ± 5.03*
t value		–0.335	–5.391
*p* value		0.738	<0.001

Note: Compared with pre-nursing status, *p < 0.05. MAAS, Mindful Attention Awareness Scale.

### Comparison of WHOQOL-BREF Scores Between the Two Groups

Analysis of extracted data showed no significant differences were observed in WHOQOL-BREF domain scores between the two groups before nursing (*p* > 0.05). Following the nursing period, extracted data indicated that scores in the physical, psychological, environmental, and social relationship domains increased significantly in both groups compared to before nursing (*p* < 0.05), and the MBSR nursing group had significantly higher scores in all four domains compared to the routine nursing group (*p* < 0.05). See Table 6.

**Table 6.** **Comparison of WHOQOL-BREF scale scores between the two groups ($\bar{x}$ ± s) point**.

Group	n	Physical domain		Psychological domain		Environmental domain		Social relationship domain	
		Before nursing	After nursing	Before nursing	After nursing	Before nursing	After nursing	Before nursing	After nursing
Routine nursing group	55	15.21 ± 2.52	17.56 ± 2.63*	10.23 ± 2.01	12.96 ± 2.12*	8.12 ± 1.53	10.72 ± 1.75*	13.23 ± 2.93	17.84 ± 3.12*
MBSR nursing group	55	15.08 ± 2.79	19.44 ± 2.71*	10.31 ± 1.95	15.63 ± 2.25*	8.07 ± 1.44	12.93 ± 1.86*	13.14 ± 3.02	20.33 ± 3.25*
t value		0.256	–3.692	–0.212	–6.405	0.176	–6.418	0.159	–4.099
*p* value		0.798	<0.001	0.833	<0.001	0.860	<0.001	0.874	<0.001

Note: Compared with pre-nursing status, *p < 0.05.

### Comparison of Polysomnography Results Between the Two Groups

Analysis of extracted data showed no significant differences were observed in polysomnography parameters between the two groups before nursing (*p* > 0.05). Following the nursing period, extracted data indicated that both groups showed significantly shorter sleep latency, fewer micro-arousals, and a higher percentage of REM sleep compared to before nursing (*p* < 0.05). Furthermore, the MBSR nursing group demonstrated significantly shorter sleep latency, fewer micro-arousals, and a higher percentage of REM sleep than the routine nursing group (*p* < 0.05). See Table 7.

**Table 7.** **Comparison of the results of polysomnography between the two groups**.

Group	n	Sleep latency (min)		REM sleep proportion (%)		Micro-arousals (times/h)	
		Before nursing	After nursing	Before nursing	After nursing	Before nursing	After nursing
Routine nursing group	55	74.85 ± 13.63	56.96 ± 14.02*	14.25 ± 2.85	16.47 ± 2.53*	38.52 ± 4.12	27.74 ± 2.86*
MBSR nursing group	55	75.08 ± 14.16	50.11 ± 10.25*	14.36 ± 2.79	18.52 ± 2.47*	39.01 ± 4.33	23.86 ± 2.41*
t value		–0.087	2.925	–0.205	–4.300	–0.608	7.694
*p* value		0.931	0.004	0.838	<0.001	0.544	<0.001

Note: Compared with pre-nursing status, *p < 0.05.

### Exploratory EFG Observations

Exploratory encephalofluctuogram (EFG) data were also extracted. It must be emphasized that EFG is not a validated tool for measuring neurotransmitter levels or brain activity; these results are purely hypothesis-generating. Detailed data are presented in **Supplementary Table 1**. In brief, the post-nursing relative power value of the 1 mHz frequency band was higher in the MBSR nursing group than in the routine nursing group (*p* < 0.05), while no significant between-group differences were observed for the other frequency bands. These observations warrant cautious interpretation and require validation with established neurochemical or neuroimaging methods.

## Discussion

In clinical practice, pharyngeal foreign body sensation, sore throat, and the degree of tonsil enlargement are intuitive indicators for assessing disease progression. Their persistence often serves as a core trigger leading to catastrophic associations and escalating anxiety in patients [30]. Analysis of the data extracted in this study indicates that, following the nursing period, patients in the MBSR nursing group had lower scores for eight pharyngeal symptoms, including dry throat, sore throat, and foreign body sensation, compared to those in the routine nursing group. Routine nursing primarily focuses on medication adherence and environmental support. In contrast, the MBSR model utilizes techniques such as the mindfulness-based cold compress which, by applying the principle of sensory channel competition-enhancing awareness of temperature and tactile sensations to inhibit pain transmission-and throat-specific body scan training, which have been associated with lower muscle tension and better local circulation [31]. Furthermore, through the mindful cold compress and S.T.O.P. short practice, the MBSR model may be associated with changes in patients’ processing pathways for pain signals. Under mindful guidance, patients anchor their attention to the physical properties of coldness rather than the pain itself. This non-judgmental awareness has been linked to a shift in attentional resources from pain-related cues to neutral somatic cues, accompanied by lower subjective intensity of pain-related experiences [32]. A randomized controlled trial by Higgins *et al*. [33] in asthma patients demonstrated that MBSR training led to clinically significant improvement in 32% of participants and reduced levels of exhaled nitric oxide, a biomarker of airway inflammation. Although the pathophysiology of chronic tonsillitis differs from that of asthma, these findings support the hypothesis that mindfulness-based approaches may promote local tissue repair by downregulating stress-related neuroimmune inflammatory responses.

Anxiety disorder is a common psychological comorbidity in patients with chronic tonsillitis [30]. The data extracted in this study show that GAD-7 and HAMA scores were significantly lower in the MBSR nursing group than in the routine nursing group after the nursing period. A randomized non-inferiority trial by Hoge *et al*. [34] found that a standardized 8-week MBSR program was comparable in effect to the first-line antidepressant escitalopram in reducing clinical global severity in patients with anxiety disorders, with a significantly lower incidence of adverse events. Although the nursing protocol documented in this study was an adapted version of the classic curriculum, it retained core techniques such as body scanning and breath anchoring, and the observed associations align with previous research findings. MBSR is thought to enhance the regulatory function of the prefrontal cortex over the amygdala, which is associated with reduced hyperreactivity to threatening stimuli [35]. Simultaneously, it may cultivate a decentered cognitive mode, allowing patients to perceive anxious experiences as temporary mental events rather than core aspects of self, which may be associated with lower intensity of emotional responses [36]. Changes in PSQI scores suggest an association between MBSR nursing and better sleep quality. The digital sunset and pre-sleep body scan practices documented in the MBSR nursing group may be associated with attenuated sympathetic excitation through parasympathetic activation, shifting attention from rumination to immediate bodily sensations, and lower pre-sleep somatic hyperarousal [37, 38]. The refined scanning of the throat area, in particular, could help patients differentiate between physiological discomfort and psychological amplification, which may contribute to lower pre-sleep somatic hyperarousal. Research by Ong *et al* .[39] also found that a mindfulness training program was associated with improved sleep quality in individuals with chronic insomnia, with reduced pre-sleep cognitive arousal identified as a contributing factor. The use of guided audio recordings in this study aligns with the convenience of mobile health, ensuring patients had a means to consistently induce relaxation responses through standardized practice even in a home environment, potentially being associated with more favorable sleep microstructure.

Research involving head and neck cancer patients suggests that MBSR is associated with reductions in depression and anxiety and may also be associated with improvements in overall quality of life through reduced stigma and social avoidance [40]. In this study, the MBSR nursing group had higher scores than the routine nursing group across the physiological, psychological, social relationship, and environmental domains of the WHOQOL-BREF and also had significantly higher MAAS scores after the nursing period. Chronic illness often leads to social withdrawal and low self-efficacy. The MBSR nursing group addressed this by establishing an online mindfulness group with a co-facilitator model and utilizing a mindfulness partner mechanism to build new social support networks. Sharing experiences of awareness may help patients reduce feelings of stigma and loneliness. This group dynamic, grounded in shared experience, could effectively foster psychological resilience. Furthermore, patients in the MBSR nursing group integrated disease management into their daily lives through mindful daily activities (e.g., mindful brushing, showering), potentially enhancing their sense of control over their health. This internal empowerment may contribute to better quality of life [41].

Regarding the exploratory EFG observations (**Supplementary Table 1**), the 1 mHz frequency band relative power was higher in the MBSR group. However, EFG is not a validated measurement tool, and these findings are hypothesis-generating only; they cannot be interpreted as reflecting neurotransmitter changes and require validation with established methods.

The extracted polysomnography data also indicate that patients in the MBSR nursing group had shorter sleep latency, fewer micro-arousals, and a higher percentage of REM sleep, which is directionally consistent with more favorable sleep microstructure parameters [39]. Physical discomfort caused by chronic tonsillitis often maintains a central hyper-aroused state during the night, resulting in sleep fragmentation. The pre-sleep cognitive defusion techniques and body scan practices documented in the MBSR nursing model may effectively counter sympathetic excitation, interrupting the vicious cycle of symptom focus, anxiety escalation, and superficial sleep. The observed lower micro-arousal frequency and higher REM sleep duration suggest an association with more stable sleep rhythms. While the mechanism remains speculative, this trajectory is directionally aligned with the exploratory EFG observations in the supplementary material, though no clinical conclusions can be drawn from the latter.

However, the findings of this study must be interpreted with caution due to several limitations. First, all primary outcomes were subjective measures, and the non-blinded design may have introduced expectancy effects that inflated the observed associations; no sensitivity analysis (e.g., E-value) was performed to assess robustness to unmeasured confounding. Second, this single-center, retrospective study had a relatively small sample, limiting generalizability. Third, although PSM was used, residual confounding from factors such as education level, marital status, and income cannot be fully excluded. Fourth, the retrospective design precludes causal inference. Fifth, only immediate post-nursing outcomes were available, with no long-term follow-up data to evaluate sustainability or relapse prevention. Sixth, MBSR intervention records relied on program-specific structured templates without independent external audit, which may limit transparency of fidelity assessment. Future prospective studies should incorporate larger samples, blinded outcome assessment, objective measures, long-term follow-up, sensitivity analyses, and independent fidelity monitoring to verify these findings.

## Conclusions

In summary, this retrospective analysis found that the MBSR nursing model was associated with lower pharyngeal symptom scores, lower anxiety levels, higher mindful attention awareness, better sleep quality, and better overall quality of life in patients with chronic tonsillitis complicated by anxiety disorder. Exploratory EFG observations are presented in the Supplementary Material and are hypothesis-generating only. Polysomnography parameters also showed more favorable values in the MBSR group. Given the limitations of the EFG methodology and the study’s retrospective design, these exploratory electrophysiological observations should be interpreted with extreme caution. Further well-controlled, prospective studies are warranted to confirm these preliminary associations and explore the potential value of this nursing model in integrated mind-body care.

## Availability of Data and Materials

All experimental data included in this study can be obtained by contacting the corresponding author if needed.
