# Supplementary material for: The Association of a Mindfulness-Based Stress Reduction Nursing Model With Quality of Life in Patients With Chronic Tonsillitis Complicated by Anxiety Disorder: A Retrospective Study
Source: Actas Esp Psiquiatr. 2026 Oct 15;54(5):1438–49. doi: 10.62641/aep.2272 (PMC13714487; doi:10.62641/aep.2272)
Supplement: Supplementary file 1 [file ActEsp-54-5-1438-1449-s1.zip › ActEsp-54-5-1438-1449.docx]

**Supplementary Materia**l

Supplementary Table S1 Comparison of Relative Power Values of Ultra-Slow Waves in EFG for the Two Groups(‾X±s) %

| Frequency band | Time | Routine nursing group（n=55） | MBSR nursing group（n=55） | t value | P value |
| --- | --- | --- | --- | --- | --- |
| 1 mHz | Before nursing | 18.99±3.02 | 19.07±2.85 | -0.143 | 0.887 |
|  | After nursing | 27.85±3.86* | 31.74±3.91* | -5.251 | <0.001 |
| 2 mHz | Before nursing | 30.63±3.71 | 30.99±3.86 | -0.499 | 0.619 |
|  | After nursing | 31.02±3.88 | 32.43±4.01 | -1.874 | 0.064 |
| 4 mHz | Before nursing | 310.12±35.21 | 305.98±37.44 | 0.597 | 0.551 |
|  | After nursing | 298.96±37.48 | 301.25±40.11 | -0.309 | 0.758 |
| 5 mHz | Before nursing | 212.41±32.56 | 208.98±35.65 | 0.527 | 0.599 |
|  | After nursing | 204.74±36.93 | 210.41±34.81 | -0.829 | 0.409 |
| 7 mHz | Before nursing | 158.63±28.51 | 162.03±27.74 | -0.634 | 0.527 |
|  | After nursing | 162.01±27.63 | 158.98±30.02 | 0.551 | 0.583 |
| 11 mHz | Before nursing | 95.23±24.41 | 93.23±26.96 | 0.408 | 0.684 |
|  | After nursing | 93.22±25.83 | 95.01±25.82 | -0.363 | 0.717 |

Note: Compared with pre-nursing status, *P<0.05; EFG, Encephalofluctuograph; The frequency band labels (e.g., 1 mHz, 2 mHz) follow conventional naming in this exploratory electrophysiological field and do not represent direct measurements of neurotransmitters. The 4 mHz band values (approximately 298–310) differ in magnitude from the other frequency bands (approximately 18–210). This discrepancy arises because the original EFG analysis software reports the 4 mHz band in absolute power units (μV²) rather than relative power percentages, consistent with the reference convention in which this band serves as a dominant reference rhythm. All values were extracted and are reported as recorded in the original reports without recalculation. The numerical values of different frequency bands are therefore not directly comparable.
